# Application of the Subcutaneous Section to the Treatment of Lipoma

**Published:** 1849-02

**Authors:** 


*Application of the Subcutaneous section to the treatment of Lipoma.*—M. Bonnet treats fatty tumours upon the above plan with success. He first introduces a sharp-pointed tenotomy knife under the tumour, and then with a probe-pointed bistoury divides the tumour upwards towards the skin, so as to reduce it to several lobules; he then squeezes the tumour so as to extravasate the fatty matter, and leaves it to be removed by absorption. This operation is repeated several times, according to the size of the tumour. No injurious effects have followed in the cases which he reports, but the results have been so favourable that he is induced to prefer the operation to the methods in general use.—***Pro. Med. and Surg. Jour., from Bulletin de Therapeutique.***

